# Increased Risk for Mother-to-Infant Transmission of Hepatitis C Virus Among Medicaid Recipients ― Wisconsin, 2011–2015

**DOI:** 10.15585/mmwr.mm6642a3

**Published:** 2017-10-27

**Authors:** Theresa Watts, Lauren Stockman, Justin Martin, Sheila Guilfoyle, James M. Vergeront

**Affiliations:** ^1^School of Nursing, University of Wisconsin–Madison; ^2^Wisconsin Division of Public Health.

State surveillance during the last 10 years reveals a nationwide increase in hepatitis C virus (HCV) infection among young adults (*1*). The proportion of infants born to HCV-infected women is also increasing nationally (*2*). To estimate the proportion of infants born to HCV-infected women and the frequency of confirmed HCV infection in their infants, maternal name and date of birth from HCV reports in the Wisconsin Electronic Disease Surveillance System (WEDSS) were linked to Wisconsin Medicaid data for 2011–2015 births. During this period, in the Wisconsin Medicaid population, the proportion of women who had evidence of HCV infection during pregnancy increased 93%, from 1 in 368 pregnancies to 1 in 192. Among 183 infants born to women with evidence of HCV viremia during pregnancy, 34% received recommended HCV testing (*3*). Mother-to-infant (vertical) transmission was documented in 4% of infants. Improvements in HCV screening practices among pregnant women and infants could enhance identification of infants at risk for vertical transmission of HCV.

Fueled by the increase in injection drug use ensuing from the opioid epidemic, the proportion of infants born to HCV-infected women is increasing nationwide (*1*,*2*). Vertical transmission is the most common mechanism of HCV infection for children, reported to occur in approximately 6% of infants born to women with HCV infection and approximately twice as often in women who are coinfected with HCV and human immunodeficiency virus (HIV) (*4*,*5*). Another risk factor that might increase the likelihood of vertical HCV transmission is presence of maternal HCV viremia (HCV RNA positivity) (*5*). Unlike other bloodborne infectious diseases that have a risk for vertical transmission, such as hepatitis B virus or HIV, for HCV there is no perinatal intervention available that has been shown to reduce vertical HCV transmission (*4*–*6*). Clinical signs of pediatric HCV infection often manifest slowly and can range in severity from being asymptomatic to fatal; liver transplantation is sometimes required (*7*,*8*). 

During 2011–2015, the reported rate of HCV among persons aged 15–44 years in Wisconsin increased 81%, from 45.7 to 82.6 per 100,000 population; 3,013 (43%) reported cases in this age group were in women (Wisconsin Division of Public Health, unpublished data, 2016). Increases in the number of women of childbearing age with HCV in Wisconsin predict an increase in the number of infants at risk for vertical transmission. The aim of this study was to estimate the proportion of women enrolled in Wisconsin Medicaid with HCV infection during pregnancy and estimate the frequency of HCV testing and infection in infants born to HCV-infected women.

Since 2000, all HCV-positive laboratory tests in Wisconsin have been reportable to the Wisconsin Department of Health Services through WEDSS. To identify maternal HCV infection, Wisconsin Medicaid encounter data for pregnant women who delivered one or more infants during 2011–2015 were extracted and linked by maternal name and maternal date of birth to WEDSS. For women who matched to both data sources, WEDSS HCV surveillance data were reviewed for evidence of HCV infection (positive laboratory reports for anti-HCV antibody or RNA). The study protocol was reviewed and approved by the Minimal Risk (Health Sciences) Institutional Review Board at the University of Wisconsin–Madison.

Consistent with a previous study (*5*), vertical transmission risk by pregnancy was classified based on presence of maternal HCV infection (anti-HCV antibody or HCV RNA). Women with HCV infection reported before their date of delivery were categorized into three risk groups: 1) high risk (evidence of viremia [RNA-positive] during pregnancy); 2) possible risk (evidence of viremia before pregnancy but no RNA results during pregnancy); and 3) unknown viremic risk (anti-HCV antibody-positive but no RNA results). Women in the cohort whose first reported HCV infection was after delivery were categorized separately, because HCV infection status during pregnancy was not known. The proportion of pregnancies at risk for vertical transmission was calculated as the number of pregnancies among Medicaid recipients who had evidence of HCV infection among all pregnancies in Medicaid recipients.

Among infants born to women at high risk, Medicaid encounter data were searched for evidence of HCV testing, indicated by a Current Procedural Terminology code or an *International Classification of Diseases, 9th and 10th Revisions*, Clinical Modification code for HCV infection from the date of birth through June 30, 2016 (last date with complete and available data). Medicaid encounter data for infants were linked by name and date of birth to WEDSS to identify evidence of HCV infection. Infants were classified as having been tested according to recommendations if the infant had an anti-HCV antibody test after age 18 months or two or more HCV RNA tests after age 2 months (*3*). HCV vertical transmission was determined through WEDSS data and was defined as a positive laboratory report of HCV infection in an infant tested for HCV per recommendations (*3*).

Among 146,267 Wisconsin Medicaid recipients who had a birth during 2011–2015, evidence of HCV infection before the delivery date was documented for 608 (0.4%) women. Among these women, 180 (30%) were classified as being at high risk, two of whom had HIV coinfection; 151 (25%) were classified as being at possible risk; and 277 (46%) were classified as having an unknown viremic risk. An additional 472 women had an HCV infection reported after their date of delivery (Figure 1). The proportion of women with an HCV infection before their date of delivery increased 93% from 2011 (2.7 per 1,000) to 2015 (5.2 per 1,000) (Figure 2); an increase from 1 in 368 pregnancies to 1 in 192.

**FIGURE 1:**
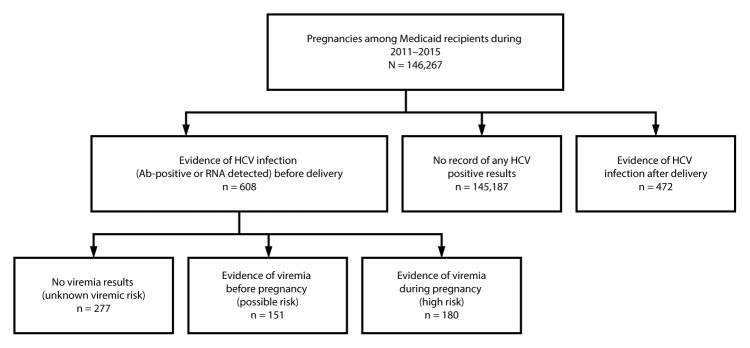
Classification of vertical transmission risk based on hepatitis C virus (HCV) infection status* among Medicaid recipients — Wisconsin Electronic Disease Surveillance System and Wisconsin Medicaid data, Wisconsin, 2011–2015 **Abbreviation**: Ab = antibody. * Women with an HCV infection reported before their date of delivery were categorized into three risk groups: women who had evidence of viremia (RNA-positive) during pregnancy (high risk), women who had evidence of viremia before pregnancy but did not have RNA results during pregnancy (possible risk), and women who were anti-HCV antibody-positive but did not have viremia results (unknown viremic risk).

**FIGURE 2:**
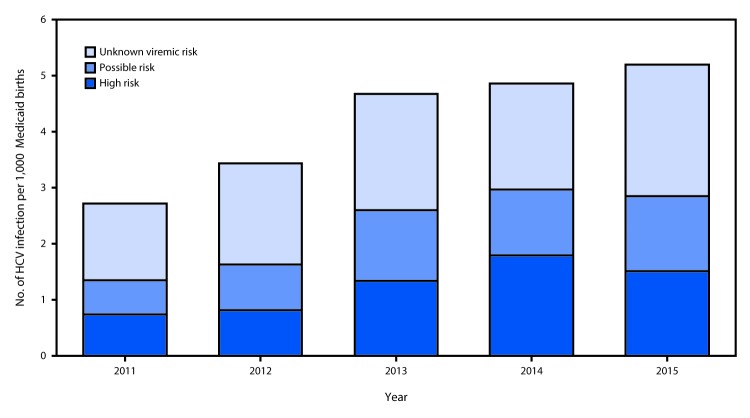
Proportion of pregnant Medicaid recipients with evidence of hepatitis C virus (HCV) infection before delivery, by risk category* — Wisconsin Electronic Disease Surveillance System and Wisconsin Medicaid data, Wisconsin, 2011–2015 * Unknown viremic risk = anti-HCV antibody-positive, but no viremia (RNA) results available; Possible risk = evidence of viremia before pregnancy, but no RNA results during pregnancy; High risk = evidence of viremia (RNA-positive) during pregnancy.

The median age of women with evidence of HCV viremia during pregnancy was 26 years (range = 18–47 years) (Table). Among the 180 women who had evidence of HCV viremia during pregnancy, 142 (79%) were non-Hispanic white, compared with 52% of women who did not have any evidence of HCV infection during pregnancy (Table). 

**TABLE:** Demographic characteristics of pregnant Medicaid recipients by hepatitis C virus (HCV) risk status — Wisconsin Electronic Disease Surveillance System and Wisconsin Medicaid data, Wisconsin, 2011–2015

Characteristic	No. (%)			
	Evidence of HCV infection before delivery	Evidence of viremia during pregnancy*	Evidence of HCV infection after delivery	No record of any HCV positive results
	(n = 608)	(n = 180)	(n = 472)	(n = 145,187)
**Race,** ^†^				
White, non-Hispanic	449 (74)	142 (79)	356 (76)	74,720 (52)
Black, non-Hispanic	48 (8)	5 (3)	14 (3)	25,398 (18)
American Indian, non-Hispanic	28 (5)	12 (7)	32 (7)	3,031 (2)
Asian, non-Hispanic	10 (2)	2 (1)	3 (0.6)	6,967 (5)
Hispanic or Latino	35 (6)	5 (3)	38 (8)	23,260 (16)
Other, non-Hispanic	18 (3)	8 (4)	8 (2)	3,028 (2)
Unknown	20 (3)	6 (3)	19 (4)	8,639 (6)
**Age group (yrs)** ^§^				
<19	19 (3)	2 (1)	58 (12)	15,937 (11)
20–29	369 (61)	123 (68)	341 (72)	91,396 (63)
30–39	200 (33)	46 (26)	71 (15)	35,569 (25)
≥40	20 (3)	9 (5)	2 (<1)	2,285 (2)
**Mean (SD)**	**28 (5.44)**	**28 (5.6)**	**25 (4.74)**	**27 (5.57)**
**Median (Range)**	**27 (17–47)**	**26 (18–47)**	**24 (14–41)**	** 25 (11–51)**

**Abbreviation**: SD = standard deviation.

* These 180 women are a subset of the 608 with evidence of HCV infection before delivery.

^†^ Whites, blacks, American Indians, and Asians were non-Hispanic; Hispanic or Latino persons could be of any race.

^§^ Mother’s age at delivery.

Among 183 infants born to women who had evidence of HCV viremia during pregnancy, 92 (50%) were continuously enrolled in Medicaid for ≥18 months (range = 18–66 months). Among these infants, 31 (34%) were tested for HCV according to recommendations, including 24 who had an anti-HCV antibody test at age >18 months and seven who had at least two RNA tests at age >2 months. Vertical transmission was documented in seven (4%) of the 183 infants born to women with evidence of HCV viremia during pregnancy.

## Discussion

Consistent with national and other state studies (*2*,*4*), these findings demonstrate that among Wisconsin Medicaid recipients, the rate of HCV infection among pregnant women is increasing. A recent national study used birth certificates to document maternal HCV infection and found that 1 in 308 infants were born to HCV-infected women in 2014 (*2*). In Wisconsin, an estimated 30% of children born to women with HCV infection do not have HCV indicated on their birth certificate (Wisconsin Division of Public Health, unpublished data, 2017). The current study which used surveillance data mandated by state statute to identify maternal HCV infection and therefore might provide more complete HCV case ascertainment, found that the rate of births to Wisconsin Medicaid-recipients with HCV infection approximately doubled from 2011 to 2015, from 2.7% to 5.2%.

The age, race, and ethnicity of women with HCV infection during pregnancy in this study were similar to those in previously reported studies (*1*,*2*,*4*) and are consistent with trends among young adults with recent HCV infection in Wisconsin (Wisconsin Division of Public Health, unpublished data, 2016). Of interest is the young age of women who had evidence of HCV infection before delivery and after delivery (median age 27 and 24 years, respectively). Without appropriate treatment for HCV, infants subsequently born to HCV-infected women are at risk for mother-to-infant transmission.

Among a subset of infants born to women with evidence of HCV viremia during pregnancy, 4% had confirmed infection. Prior studies have indicated a lack of adequate HCV testing among children born to HCV-infected women (*4*,*9*). In the current study, only 34% of Wisconsin Medicaid-recipient infants born to women with evidence of HCV viremia during pregnancy were tested for HCV according to recommendations (*3*), revealing a substantial gap in monitoring infants at risk for HCV vertical transmission.

The findings in this report are subject to at least four limitations. First, statewide surveillance data were used to identify HCV infection status and vertical transmission risk category. These data rely on reports from risk-based HCV testing and laboratory reporting and are likely to underestimate the number of women and children with HCV infection. Second, HCV RNA–negative results were not reportable at the time of analysis. Therefore, the number of women with resolved HCV infection is unknown. However, because there are no approved treatment regimens for HCV during pregnancy, it is unlikely that women classified as at high risk had resolved infection before delivery. Third, only 50% of infants born to women at high risk were continuously enrolled in Medicaid, and therefore, HCV testing data for all infants were unavailable. Finally, this analysis of women and infants enrolled in Medicaid represents approximately 38% of the deliveries in Wisconsin during the study period.*

 Enhanced identification through HCV screening during pregnancy and public health follow-up to monitor infants at risk for vertical transmission are needed. The current recommendation for identifying HCV-infected pregnant women is through risk-based screening (*3*,*10*). Pregnancy and postpregnancy care might provide an opportune time to test women and link HCV-infected women to HCV care or treatment, because this is a time when a woman might be likely to use health care services. To improve surveillance of HCV vertical transmission, support identification of cases, and evaluate health outcomes of infected infants, the Council of State and Territorial Epidemiologists^†^ recently approved of and issued a position statement for reporting and national notification of perinatal HCV infection. Adoption of this position statement by state and local health departments, along with enhanced identification of HCV among women of childbearing age, can improve care for HCV-infected women and infants at risk for HCV vertical transmission

**Summary**

**What is already known about this topic?**

Nationally, the number and rate of hepatitis C virus (HCV) infections among women of childbearing age has increased, suggesting that the number of infants born to HCV-infected women has also increased.

**What is added by this report?**

Among Wisconsin Medicaid recipients, the rate of HCV infection during pregnancy is increasing. During 2011–2015, the proportion of women who had HCV infection before their date of delivery increased 93%, from 1 in 368 pregnancies to 1 in 192 pregnancies. Among the infants born to women who had evidence of HCV viremia during pregnancy, 34% received HCV testing per the recommendations and evidence of vertical transmission was documented in 4% of infants.

**What are the implications for public health practice?**

As the rate of HCV infection among women of childbearing age continues to increase nationally, practices for screening pregnant women for HCV and for monitoring infants born to HCV-infected mothers should be improved. Enhanced identification through testing all pregnant women with HCV risk factors and improved public health surveillance of infants at risk for HCV vertical transmission will improve identification, detection, and care for HCV-infected women and infants at risk for HCV vertical transmission.

## Footnotes

* https://www.dhs.wisconsin.gov/wish/birth/form.htm.

† http://c.ymcdn.com/sites/www.cste.org/resource/resmgr/2017PS/2017PSFinal/17-ID-08.pdf.
